# Impact of a deferred recruitment model in a randomised controlled trial in primary care (CREAM study)

**DOI:** 10.1186/s13063-017-2284-x

**Published:** 2017-11-10

**Authors:** Victoria Shepherd, Emma Thomas-Jones, Matthew J. Ridd, Kerenza Hood, Katy Addison, Nick A. Francis

**Affiliations:** 1 Centre for Trials Research, Cardiff University, Cardiff, UK; 2 Division of Population Medicine, School of Medicine, Cardiff University, Cardiff, UK; 3 School of Social and Community Medicine, University of Bristol, Bristol, UK

**Keywords:** Primary care, Randomised controlled trial, Clinical trial recruitment, Recruitment challenges, Informed consent

## Abstract

**Background:**

Recruitment of participants is particularly challenging in primary care, with less than a third of randomised controlled trials (RCT) achieving their target within the original time frame. Participant identification, consent, randomisation and data collection can all be time-consuming. Trials recruiting an incident, as opposed to a prevalent, population may be particularly affected. This paper describes the impact of a deferred recruitment model in a RCT of antibiotics for children with infected eczema in primary care, which required the recruitment of cases presenting acutely.

**Methods:**

Eligible children were identified by participating general practitioners (GPs) and referred to a study research nurse, who then visited them at home. This allowed the consent and recruitment processes to take place outside the general practice setting.

Information was recorded about patients who were referred and recruited, or if not, the reasons for non-recruitment. Data on recruitment challenges were collected through semi-structured interviews and questionnaires with a sample of participating GPs. Data were thematically analysed to identify key themes.

**Results:**

Of the children referred to the study 34% (58/171) were not recruited – 48% (28/58) because of difficulties arranging a baseline visit within the defined time frame, 31% (18/58) did not meet the study inclusion criteria at the time of nurse assessment, and 21% (12/58) declined participation. GPs had positive views about the recruitment process, reporting that parents valued and benefitted from additional contact with a nurse. GPs felt that the deferred recruitment model did not negatively impact on the study.

**Conclusions:**

GPs and parents recognised the benefits of deferred recruitment, but these did not translate into enhanced recruitment of participants. The model resulted in the loss of a third of children who were identified by the GP as eligible, but not subsequently recruited to the study. If the potential for improving outcomes in primary care through complex studies is to be realised, new approaches to recruitment into primary care trials need to be developed and evaluated.

**Trial registration:**

International Standard Randomised Controlled Trials, ISRCTN96705420. Registered on 27 June 2012.

## Background

Randomised controlled trials (RCTs) conducted in primary care settings are essential for generating the evidence base required for general practice, where the majority of healthcare is provided in the UK. However, complex studies, such as RCTs, are often challenging and time-consuming for participating sites, and research projects across all settings are frequently impacted by poor recruitment [1]. A review of multicentre trials found that problems with recruitment were common, complex and challenging [2]. However, it is particularly an issue in primary care, as shown by a survey of UK primary care studies which found that significant recruitment difficulties were encountered, with less than a third of studies recruiting to their original timescale [3]. This was despite a variety of methods being used to identify and recruit participants, such as advertisements and screening in waiting rooms, and systematic identification of eligible patients from practice records [3]. Barriers encountered in RCTs in primary care settings, and strategies and facilitators used to improve patient recruitment, have been widely reported [3–5].

Trial recruitment processes vary by research questions and the condition being investigated. Trials of preventive strategies which recruit healthy participants will pose different recruitment challenges than trials into the management of patients with chronic conditions, which in turn are quite distinct from studies in acute and acute-on-chronic conditions [1]. The comparative difficulty of recruiting an incident, as opposed to a prevalent, population – which necessitates opportunistic clinician referrals of patients with acute conditions – exacerbates the problem of recruitment in primary care [6]. Insufficient time for general practitioners (GPs) to raise and invite participation to a trial, receive informed consent, and complete documentation during a consultation, has been perceived as a major barrier [3, 7, 8].

A novel approach is the ‘deferred recruitment model’ (distinct from delayed consent in emergency situations) adopted in the CREAM (Children with eczema, antibiotic management) study. The CREAM study was a RCT that aimed to determine the effectiveness of orally and topically administered antibiotics, in addition to standard treatment with emollients and topically administered corticosteroids, on subjective and objective eczema severity in children with clinically infected eczema in primary care [9]. The deferred recruitment model allowed the informed consent and recruitment procedures and baseline data collection to take place at a later time in the participant’s home, rather than during the consultation. It was intended to minimise the challenges of in-consultation recruitment and to reduce the impact for parents and young children of a prolonged surgery visit. Whilst many approaches to recruitment in primary care research have been described [10] a deferred recruitment model has not previously been reported [11].

Better understanding of facilitators and barriers to recruitment, including the impact of trial design, is essential to develop effective approaches to recruitment [12].

The gap in knowledge and evidence for effective recruitment strategies has been reported, alongside the recommendation for trialists to include evaluations of their recruitment strategies in order to improve the conduct and efficiency of future trials [13]. Evidence from recruitment methods used in practice, such as presented here, may be helpful when planning similar studies, together with the development of an evidence-based, practical framework for recruitment in primary care [14].

## Methods

Details of the CREAM study are described elsewhere [9]. General practices (*n* = 95) and dermatology clinics (*n* = 4) were recruited as sites. Potentially eligible children with a history of eczema were identified by GPs, or in primary care dermatology clinics, from their patient lists and their parents were informed by letter that the practice was taking part in the study. If the child presented with suspected infected eczema, and the GP confirmed that they were eligible for the study (Appendix: Inclusion and exclusion criteria) their parents received further information, including the Participant Information Sheet. If they were willing to take part, the GP referred them to the local CREAM study research nurses. GPs completed a referral proforma and a prescription for the trial medication which the parent signed to indicate that they were willing to be referred. These were faxed to the co-ordinating centre and the designated clinical trial pharmacy, respectively. The child was also prescribed a moderately potent topically administered corticosteroid and an emollient.

Research nurse support was provided from research networks and research nurses employed specifically for the trial. Research nurses arranged a baseline visit to the participant’s home within a specified time frame. Due to concerns about children with infected eczema waiting to be seen by the research team, this was initially 48 h from referral by the identifying site. However, concerns about complications within this time frame were not realised and in order to be able to recruit patients identified on a Friday and optimise recruitment, the maximum time was increased to 72 h from receipt of the referral. However, the aim was to visit most children within 24 h. Potential participants were randomised to one of three study arms by a study pharmacy: orally and topically administered placebos, orally administered flucloxacillin and topically administered placebo, or topically administered fusidic acid and orally administered placebo, for 1 week.

The baseline visit by the research nurse included providing information about the study, checking eligibility, obtaining informed consent, completing a clinical assessment and taking swabs, completing questionnaires, and arranging follow-up visits. The research nurse would collect the trial medication from the clinical trials pharmacy prior to the baseline visit, and would release this to the parent following provision of informed consent with an explanation of how to use the treatments. Participating general practices were spread across wide geographical areas, which necessitated research nurses travelling from their base (or previous participant visit), to the centralised pharmacy which dispensed the trial medication, and then out to the participant’s home.

The baseline visit took up to 2 h to complete, and it was thought that young children may be more at ease and compliant with clinical assessments if this was undertaken in their home environment. It was anticipated that a visit to the child’s home would be more convenient for parents, which would likely be considered a positive feature of the study when GPs were inviting parents to take part.

The delay from referral to recruitment of up to 72 h necessitated a confirmation at the time of consent that the child’s eczema had not worsened, requiring an urgent review of their management, or that any other change had occurred that rendered them ineligible. A referral faxed by a GP surgery after ‘office hours’ was received by the trial team the following morning, which may mean the baseline visit occurred early on the 4th calendar day in some cases. It was the referring clinician’s responsibility to decide whether it was safe and appropriate to refer a child on a Friday, knowing they would not be seen by a research nurse over the weekend. Follow-up visits at 2 and 4 weeks were also conducted in the participant’s home usually by the same research nurse, with 3-month follow-up conducted via parent-completed postal questionnaire and swabs and a primary care medical records search.

Participating clinicians were asked to keep screening logs to record information about patients referred to the study team. Research nurses also recorded details about whether patients referred to the study team were recruited and, if not, the reasons for non-recruitment. Data were not recorded on the number of attempts made to contact parents, the time period between referral and contact by the research nurse, or the time between referral and a decision not to participate.

Data on recruitment challenges were collected through a combination of semi-structured, audio-recorded telephone interviews and semi-structured questionnaires. We identified a purposive sample of general practices that had recruited the highest numbers of participants (recruited three or more participants) (*n* = 4), low recruiting practices (recruited one to three participants) (*n* = 6), and practices that were engaged but had not recruited any participants (*n* = 3), across English and Welsh sites. The principal investigator (a GP) at each site was contacted and invited to participate in a short telephone interview. Those who we were not available for interview were invited to complete a semi-structured questionnaire to be returned by post or email.

Those practices that reported that they were unable to respond formally (due to time or other resource issues) were asked to provide informal feedback either by email or telephone conversation. This informal feedback supported the findings from analysis of the interview and questionnaire data. Research nurse field notes, regular documented meetings and opportunistic feedback from GPs and other stakeholders were also used as data sources.

An interview topic guide defined the main topics, whilst allowing flexibility to pursue issues in more depth as they emerged from the interviews. Interviews were audio-recorded and transcribed verbatim. We used post hoc descriptive analysis to evaluate the impact of the recruitment model.

## Results

**Fig. 1:**
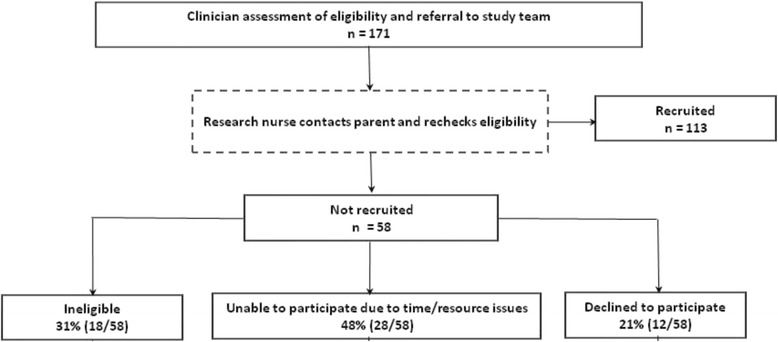
Participants referred and not recruited. Children were assessed by a clinician for eligibility for the trial, and were subsequently either recruited or not recruited to the trial. Data are shown as number of children who were assessed, and recruited or not recruited. Data for children not recruited are shown divided by category, and expressed as a percentage of children not recruited

Of the 95 sites that were initiated only 35% (32 GP sites and one dermatology clinic) actively recruited one or more participants into the study. A total of 171 children were identified by participating clinicians and referred to the study, and of these, 113 (66%) were recruited. Four patients (4%) were recruited from primary care dermatology clinics, 109 (96%) came from general practices. The median referral to recruitment interval was 1 day (57% same day, 12% 1 day, 10% 2 days, 7% 3 days, 14% 4 days). Of the 34% (58/171) of patients referred to the study who were not recruited, all were referred from general practices (Fig. 1).

### Ineligibility of children

Of the children who were referred but not recruited, 31% (18/58) were identified by the research nurse as not meeting the study inclusion criteria either at the initial telephone consultation (94% 17/18) or at the baseline visit (6% 1/18) prior to consent and registration.

The most frequent reason for ineligibility was the recent use of antibiotics or potent or very potent corticosteroids, which accounted for 15% (9/58) of those referred by clinicians but not recruited.

### Unable to participate due to time and resource issues

Almost half of those not recruited were because of difficulties in arranging a baseline visit 48% (28/58). This included cases where parents were either not contactable by the research nurse (29% 8/28) or were unavailable for a baseline visit for unknown reasons (11% 3/28). Of children referred and not recruited, 16% (9/58) were not recruited because the research nurse was unable to arrange the baseline visit within the agreed time frame as the parents were not available. Reasons provided for the unavailability of the parent included work commitments (78% 7/9), family holidays (11% 1/9), and the child being in school or childcare during the period that a baseline visit would need to be conducted within (11% 1/9). An additional 29% (8/28)) of children were not recruited because research nurses reported a lack of time or resources. Where reasons were provided, the main reasons given for research nurse unavailability were conflicting study appointments (25% 2/8), and the amount of time required to allow for dispensing and collection of the study medication from the hospital pharmacy prior to the visit (25% 2/8).

### Parents declined participation

Of those children who were referred but were not recruited, 21% (12/58) were not recruited because their parents decided that they did not wish to participate. More than a third (42%, 5/12) of those who did not wish to take part cited that they were not willing to use antibiotics as the reason for their decision. These parents did not appear to have a complete understanding of the study when they were referred by their clinician, and declined upon receiving information that the study was evaluating the use of antibiotics. Two parents (17% 2/12) indicated that they had changed their mind about participating in the study. Two parents (17% 2/12) reported that the child’s eczema had improved from the initial consultation to an extent that they no longer wished to take part in the trial. This improvement was attributed to the use of standard steroid and emollient care during the period from referral to contact by the research nurse.

### Views of participating GPs

Eight GPs from practices with high recruitment (2), low recruitment (3) and no recruitment (3), contributed qualitative data (three interviews, five semi-structured questionnaires). Three GPs felt very positively about the research nurse visit as part of the study design, which was described as an ‘important’ aspect. Several GPs reported that they had received feedback from parents that they liked the home visit, which they perceived as ‘specialist care in their own homes’, particularly when the research nurse had a dermatology background. Parents felt that they benefitted from additional contact with a knowledgeable healthcare professional that they would not have received if they had not participated in the study. Participating GPs reported that the deferred recruitment model did not impact negatively on the study. One practice reported that having more research nurse time available would have been beneficial to recruitment at their practice.

The additional administrative burden that resulted from referral to the study team for deferred recruitment was explored. The requirement to complete two prescriptions, a routine prescription for a corticosteroid and an emollient, and a study-specific prescription for study medication (which was then faxed to the site pharmacy) was reported as problematic by several GPs. The plan had been for study documents to be transmitted electronically, but we encountered significant problems with the specialist software designed to do this, and the software was eventually withdrawn, necessitating the use of fax to transmit documents. This was particularly burdensome as the study prescription needed to be faxed to the site pharmacy and other documents needed to be faxed to the study team. GPs did not think that recruitment being conducted outside the practice by external personnel had reduced practice ‘buy in’ to the study, although a lack of awareness about the study among the practice team was commonly found during research team visits and telephone contact with practices.

Informal feedback obtained from participating GPs and practices supported these findings, with practices commonly citing the low numbers of children consulting with suspected infected eczema and the difficulty of defining infection and eligibility as resulting in low rates of recruitment.

### Management of resources

The trial was resource intensive, requiring significant levels of research nurse support. The balancing of opportunistic, unplanned referrals, narrow time windows for completing baseline visits, the time required for dispensing study medication, and travel time to a participant’s home often made it difficult to arrange baseline appointments. Our recruitment rate was lower than anticipated and unplanned recruitment visits needed to be arranged at short notice; therefore, balancing research nurse workflow and available personnel was challenging. As different models of research nurse support were used in different regions, requirements and available support were determined for each region, and required a degree of flexibility and modification.

Difficulties arose when referrals were received from clinicians prior to the weekend or public holidays. Research nurses endeavoured to contact parents at the earliest opportunity. Out-of-hours’ visits were rarely possible due to the restriction of clinical trial pharmacy opening times.

## Discussion

The deferred recruitment model – using research nurses to recruit participants in the days following identification in general practice – helped reduce the burden for GPs, but may have hindered recruitment potential in other ways. Whilst it helped to address the issue of practice staff not having the time or flexibility to be able to conduct the 2-h baseline assessment, which would also require training staff in multiple practices to conduct assessments, it resulted in loss between identification and recruitment and may have deterred some parents from participating. These events may have been less likely if the child was recruited into the study in the practice on the day of the consultation.

It is difficult to compare recruitment rates in this trial and other trials in primary care due to the distinctive features of the trial – a clinical trial of a medicinal product in a paediatric population, requiring recruitment during incidence of infection in a primary care setting. Alternative models of recruitment used in prevalence or registry-based studies, such as prior parental consent for children diagnosed with eczema in participating practices or self-referral, could have been considered. However, neither of these approaches are likely to have been feasible in this study. Consenting all children with eczema would have been problematic, as only a very small proportion would have been likely to become eligible during the trial period. Similarly, self-referral to the trial would have been problematic given the need for the diagnosis to be confirmed clinically, and the need for prescribed corticosteroid and emollient treatment and trial medication.

Arranging a baseline visit at a convenient time for both parents and study personnel, and within the time frames specified in the protocol, was problematic. Parents’ willingness to be referred to the study team did not necessarily reflect their intention to participate in the trial, which may have been due to an incomplete understanding about the study. It has been reported elsewhere that there has been a shift from parents’ expectation and demand for antibiotics when their child is unwell to concerns regarding antibiotic resistance related to unnecessary prescribing [15]. This may be a result of awareness campaigns about the links between antimicrobial use and antimicrobial resistance [16].

We were limited to practices that were within travelling distance of research nurses and clinical trials pharmacies. The short shelf-life of the oral preparations once reconstituted (7 days) required same-day delivery to trial participants. However, dispensing procedures meant that participating clinical trials pharmacies required a minimum of 2–4 h notice prior to a baseline visit. This time was longer than for routine dispensing of orally administered antibiotics as clinical trial pharmacies are based in large teaching hospitals that commonly experience high dispensary workloads, and additional documentation and processes are required when dispensing an investigational medicinal product. This was a major challenge in terms of recruiting practices and participants, and led to difficulties in expanding the study.

Ethical and methodological concerns may have been raised about randomisation and allocation of the trial medication occurring prior to written consent to participate (although the parent had consented to their child being referred to the study); however, these concerns were not realised. No concerns were raised by the Research Ethics Committee during their review, and a favourable ethical opinion was obtained. The trial design ensured that the research nurse only released the trial medication once informed consent had been obtained and a consent form signed. At the conclusion of the trial, it remained sufficiently balanced in terms of allocation between trial arms [9].

In terms of health and well-being concerns, there were only two incidents of children who required further consultation with their GP between referral and recruitment, and were subsequently prescribed antibiotics and not recruited. Therefore, the recruitment method is unlikely to have affected our measure of treatment effect. No serious adverse events were recorded during the trial.

### Limitations

We did not evaluate the model as part of a formal randomised evaluation of its effectiveness. A formal process evaluation or qualitative analysis did not form part of the original study design, so the findings presented here only report on available data. As practices did not reliably record details of potentially eligible children screened or reviewed in their practices, there are no data regarding children who consulted their GP for infected eczema and were not referred because they were ineligible at the time of consultation or whose parents were not interested in taking part. Due the comparatively small number of children recruited from dermatology clinics, comparisons between the different models of recruitment in the two settings cannot be made.

Reasons recorded for referred children not being recruited are limited in scope, with no reason recorded for the ineligibility or non-participation of some children, in some cases perhaps because the reason was not known or as a result of detailed reasons not being recorded where contact was made. There are many possible reasons that the parents of eight of the children may have been uncontactable, including that they decided not to participate. Reasons for deciding not to participate may have included discussion with another person, improvement of the child’s eczema, or other treatment being sought following referral. Some reasons for not participating, such as due to an incomplete understanding of the study, may have occurred regardless of whether there was a delay in recruitment or not.

As there are no data regarding the eczema severity or extent for children who were not recruited and, therefore, did not provide baseline data, the characteristics of participants versus non-participants cannot be compared. There may have been GP-specific effects on recruitment or non-recruitment of children presenting, including their degree of equipoise, the quality of information provided to potential participants, and any differential effects on the application of eligibility criteria. We did not record the time period between referral and initial contact by the research nurse; therefore, we cannot determine if there is an association between the length of time between contact and whether the child was recruited or not.

A relatively small number of number of GPs were involved in the evaluation. The GPs participating in interviews and returning questionnaires were more likely to be engaged with the study and responsive to approaches from the study team. This may have affected clinician and parent perception of the study in those practices as a consequence.

GPs’ views on alternative recruitment models, such as GPs obtaining informed consent during the consultation with research nurses conducting the baseline assessments at a subsequent appointment, were not explored.

### Recommendations

With increasing demands on primary care and a shortage of clinicians, alternative strategies to minimise the burden of recruitment and consent processes for GPs are urgently required. Although deferred recruitment was seen as acceptable and welcome by the GPs participating in our evaluation, a large proportion of patients identified and referred in to the study were lost. Although participants seemed to value the additional time and expertise that a nurse was able to give them, this could perhaps be done alongside a GP consultation in a practice setting. GP surgeries may have found it difficult to conduct a baseline appointment during the consultation, given the time required to complete all the assessments. However, GPs obtaining informed consent during the consultation, followed by referral to the research nurse for a subsequent baseline assessment visit, may have improved the recruitment process. The follow-up visits being conducted by research nurses in the participants’ homes at 2 and 4 weeks may confer the benefit of ongoing eczema advice that was generally considered as a facilitative factor by both GPs and parents, without restriction from the narrow time frame required at the time of recruitment.

Methods that enable primary care studies to recruit opportunistically during consultations need to be developed, especially for low-risk comparative effectiveness studies that compare existing licensed technologies/products. Recent primary care trials have demonstrated that it is possible to both recruit and randomise young children with eczema during a consultation, but that retention rates may be lower [17] and recruitment more problematic [18] using in-consultation pathways when compared with other models. Consent procedures and documentation could be simplified for pragmatic studies involving medicines with a well-established safety profile. However, the ethical, regulatory and practical issues would require careful exploration. Much of the baseline data collected are already collected by clinicians and would be relevant to their clinical assessment and medical record keeping. Systems to facilitate the electronic capture of this information and integrate it with the medical record would be of value to clinicians and researchers. Further exploration of recruiting clinicians and participants’ views on alternative models of consent and recruitment, such as deferred recruitment, in other conditions and with different study designs is required. Further assessment of this model through a randomised evaluation may be warranted.

## Conclusions

Several recruitment issues were encountered in this study, including the loss of potential participants in primary care between identification and recruitment. A key decision in the design of the trial was to have research nurses visit potential participants at home in order to minimise the burden for participating GPs to gain consent, which had been found elsewhere to be a barrier to recruitment.

The requirement to complete the baseline visit within a short time frame, to maintain the safety of participants and provide accurate baseline data, was a barrier. Despite research nurses offering flexible appointments, difficulties arranging baseline visits remained if the child was of school age and well enough to attend school, or parents had work commitments, due to the length of time needed for a baseline visit and the need to assess the child’s skin and take swabs during the visit.

Alternative strategies to minimise the burden of recruitment and consent processes for GPs, particularly during routine consultations, are urgently required in order to address the challenges of recruitment to clinical trials in primary care.

## Appendix

### Inclusion and exclusion criteria

Children were eligible to join the study if they met the following inclusion criteria and did not meet any of the exclusion criteria:

**Table:**

Inclusion criteria:
Children (aged 3 months to less than 8 years) with atopic eczema who presented with a clinical suspicion of infected eczema. This could include children where:
The eczema was failing to respond to standard treatment with emollients and/or mild-to-moderately potent topically administered corticosteroids
There was a flare in the severity or extent of the eczema
There was weeping or crusting
Exclusion criteria
Children were not eligible for inclusion if they had:
Used orally or topically administered antibiotics to treat a skin infection within the past week
Used potent or very potent topically administered corticosteroids within the past 2 days
Features suggestive of eczema herpeticum (significant pain, punched out lesions)
Known significant comorbid illness (e.g. significant immune compromise)
Allergy to fusidic acid or both penicillin and erythromycin
Contraindication to any study medication (penicillin, erythromycin, fusidic acid)
A treating clinician that believed the patient had a severe infection requiring immediate antibiotics or was arranging immediate hospitalisation or urgent (same or next day) dermatology referral because of the severity of the eczema or suspected infection
A parent/legal guardian was unable to provide written informed consent
A parent/legal guardian (or a person delegated by the parent/legal guardian) was not available for follow-up visits and who did not understand English well enough to complete verbal and written questionnaires

## Abbreviations

GP: General practitioner
RCT: Randomised controlled trial
